# Significant variations in Weber fraction for changes in inter-onset interval of a click train over the range of intervals between 5 and 300 ms

**DOI:** 10.3389/fpsyg.2014.01453

**Published:** 2014-12-12

**Authors:** Pekcan Ungan, Suha Yagcioglu

**Affiliations:** ^1^Department of Biophysics, School of Medicine, Koç University, Istanbul, Turkey; ^2^Science Academy, Istanbul, Turkey; ^3^Department of Biophysics, Faculty of Medicine, Hacettepe University, Ankara, Turkey

**Keywords:** interval timing, pitch perception, internal clock, JND, Weber’s fraction, EEG alpha rhythm

## Abstract

It is a common psychophysical experience that a train of clicks faster than ca. 30/s is heard as one steady sound, whereas temporal patterns occurring on a slower time scale are perceptually resolved as individual auditory events. This phenomenon suggests the existence of two different neural mechanisms for processing of auditory sequences with fast and slow repetition rates. To test this hypothesis we used Weber’s law, which is known to be valid for perception of time intervals. Discrimination thresholds and Weber fractions (WFs) for 12 base inter-click intervals (ICIs) between 5 and 300 ms were measured from 10 normal hearing subjects by using an “up–down staircase” algorithm. The mean WF, which is supposed to be constant for any perceptual mechanism according to Weber’s law, displayed significant variation with click rate. WFs decreased sharply from an average value of around 5% at repetition rates below 20 Hz to about 0.5% at rates above 67 Hz. Parallel to this steep transition, subjects reported that at rates below 20 Hz they perceived periodicity as a fast tapping rhythm, whereas at rates above 50 Hz the perceived quality was a pitch. Such a dramatic change in WF indicated the existence of two separate mechanisms for processing the click rate for long and short ICIs, based on temporal and spectral features, respectively. A range of rates between 20 and 33 Hz, in which the rate discrimination threshold was maximum, appears to be a region where both of the presumed time and pitch mechanisms are relatively insensitive to rate alterations. Based on this finding, we speculate that the interval-based perception mechanism ceases to function at around 20 Hz and the spectrum-based mechanism takes over at around 33 Hz; leaving a transitional gap in between, where neither of the two mechanisms is as sensitive. Another notable finding was a significant drop in WF for ICI = 100 ms, suggesting a connection of time perception to the electroencephalography alpha rhythm.

## INTRODUCTION

It is known that a train of clicks at fast rates (above 30–40 clicks/s) is perceived as one continuous sound, whereas temporal patterns occurring on a slower time scale are perceptually resolved as individual auditory events (Fritz et al., 2005). This psychoacoustic observation and many others differentiating between the high (e.g., spectral, analytical, tone, or place) pitches and the low (e.g., periodicity, virtual, repetition, or residual) pitches suggest a transition from a frequency-based perception mechanism for continuous sounds, to an interval-based perception mechanism for sequences of individual auditory events. One of the ways to find out if there are two separate mechanisms for processing slow and fast click rates would be to determine the just noticeable changes in inter-click interval (ICI) in these two cases and compare the corresponding Weber fractions (WFs) to see if they differ significantly. This approach is based on a well-known psychophysical rule called Weber’s law which states that the just-noticeable change (Δ*I*) in the magnitude of a stimulus is proportional to the magnitude *I* of the stimulus prior to the change (Gescheider, 1997). In other words, the ratio Δ*I*/*I*, which is called the WF should be constant and regarded as a characteristic feature of the perceptual system processing the stimulus.

According to the widely accepted scalar expectancy theory (SET), which is based on an internal clock-counter dedicated to measuring the passage of time, the variability of temporal estimates increases proportionally with the magnitude of time (see Gibbon et al., 1984; Wearden, 2005). This means that Weber’s law holds for time perception, and WF should remain constant over a wide range of durations. If this argument is applied to the mechanism that perceive the ICI in a click train, the ratio ΔICI/ICI, where ΔICI is the just noticeable change around a base ICI value, should remain the same as ICI changes. Of course this is true if a single perception mechanism covers the whole range of ICIs studied. And, any significant alteration in WF in a particular range of ICIs could be taken as evidence for the involvement of another time perception mechanism in that range. In fact, there are quite a number of studies in literature demonstrating disruptions in psychophysical functions and violations of Weber’s law in time perception (see the reviews by Grondin, 2001; Lewis and Miall, 2009). The reader is also referred to the work of Gibbon et al. (1997) where pooled data from 19 different human studies are presented. These pooled data suggest a gradual increase in WF with longer intervals and a break point at approximately 100 ms and perhaps another at 1.5 s. However, in order to better characterize the behavior of the WF, we performed the present study in which a single task was used across a broad range of intervals between 5 and 300 ms, which covers the ICI range where a perceptual transition from a homogenous sound to a pulsed sound (or vice versa) is known to occur. Our aim was to investigate how this perceptual transition range compares with the interval range in which a brake occurs in the ICI differentiation threshold (WF).

Weber fractions for detecting a change in repetitive auditory stimulation intervals longer than 67 ms (i.e., stimulus rates lower than 15 Hz) have been well documented in studies on tempo perception (e.g., Michon, 1964; Drake and Botte, 1993; Westheimer, 1999; for reviews, see Grondin, 2001; McAuley, 2010). There are also many studies on pitch discrimination threshold for sound frequencies higher than 100 Hz (e.g., Demany and Semal, 2002; Tramo et al., 2002; Bernstein and Oxenham, 2003; Tervaniemi et al., 2005). However, we were able to find only two studies (Krumbholz et al., 2000; Phillips et al., 2012) in the literature on the rate discrimination thresholds and/or WFs for the click repetition rates we are interested in; i.e., the range of rates in which the above mentioned psychoacoustic transition from perceiving a single steady sound to perceiving each one of the individual click sounds in a train takes place. The data reported in the former one of these studies for a number of repetition rates between 16 and 256 Hz exhibit, indeed, a rapid, 10-fold decrease in the relative rate discrimination threshold (i.e., WF for differences in click rate) as repetition rate is increased from 16 to 64 Hz. Interestingly, the authors also reported that the perceptual quality of the stimuli changed from flutter to pitch over this range. A similar sharp drop in the WF as the intervals between broadband clicks is decreased form 40 to 10 ms (as the rate increases from 25 to 100 Hz) is also seen in the curves provided in the latter of these studies for a number of rates between 10 and 200 Hz.

In the study of Krumbholz et al. (2000), the minimum click rate employed was 16 Hz. Phillips et al. (2012) extended in their study the range of click rate but only down to 10 Hz (for ICI = 100 ms) and they observed a notably sharp drop in WF at this end. However, it is not clear how the “WF–click rate” relationship would extend toward even slower repetition rates where individual clicks are more distinctly perceived. A part of this missing range of relatively low click rates was studied down to 3.3/s in the present work. The higher rates which had already been studied in the above-mentioned works were also included. In this way, the tests in a wider range of click rates were all conducted on the same cohort of subjects and by using the same methodology and instrumentation. Therefore, alterations in WF could be assessed with no inter-group or inter-lab variance, which might have occurred if the data from different laboratories were stitched together for this purpose.

## MATERIALS AND METHODS

Ten volunteers (six males) with normal hearing took part in the study. The ages of the subjects ranged from 20 to 42 years with a median of 24 years. Experimental procedures were conducted in accordance with the Declaration of Helsinki and were approved by the Institutional Research Ethics Committee of Koç University (Nr.: 2013.176.IRB2.53). Informed written consent was obtained from all participants prior to the experiment and they were paid for their time.

Determination of the region of click rate where the perceptual transition mentioned above occurs was achieved by comparing the relative ICI discrimination thresholds (WFs) for 12 different base ICI values between 5 and 300 ms, corresponding to base repetition rates between 200 and 3.3 Hz, respectively. Click sounds were generated under Matlab R2012a (The MathWorks, Inc., Natick, MA, USA) using a PC and a sound card (Creative, SB-1095) with a sampling rate of 80 kHz and 24-bit precision. Binaural stimuli were delivered via an audiological insert earphone set (Etymotic, E.A.RTONE-3A). The electrical waveform of a click was a single period (0.1 ms) of a full sine wave. For the rates up to 50 Hz the intensity of the clicks is adjusted to 60 dB(nHL) referenced to the average detection thresholds of five of the normal hearing subjects when they listened to a single click. For maintaining comfort of the subjects, intensity of the clicks was reduced by 10 dB for the rates 50 Hz and above in order to compensate for the rise in sound energy and subjective intensity at relatively high rates.

**FIGURE 1:**
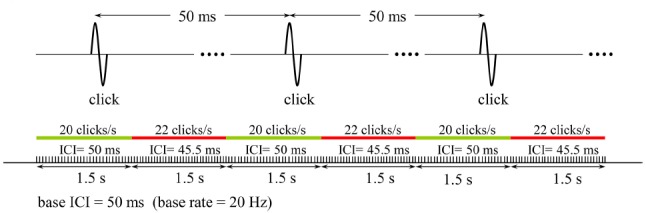
**The stimulation pattern that was used in the psychophysical tests to determine the detection threshold for a change in the base rate of a click train (20 Hz in this example).** Subjects responded by pressing 1 or 0 on a keyboard to indicate if they detected or not the change, respectively. The relative change in click rate (10% in this example) was decreased or increased depending on whether the response was positive or negative, and the test was terminated when five reversals in the response were recorded. The same test was repeated for all the 12 base rates studied, and the variation of Weber fraction with click rate was determined.

A 6-s train of clicks, of which the ICI were alternated every 1.5 s between a base value and an ICI somewhat shorter than that, were presented to the subject and he/she was asked to indicate whether he/she had detected the modulation in ICI. In the example schematically illustrated in Figure 1, the base and the shorter ICIs are 50 and 45.5 ms, respectively. By using such a paradigm for discrimination threshold testing, rather than presenting the two click rates with a gap in between and ask the subject to compare them, we tried to avoid substantial involvement of working memory for comparisons.

Discrimination thresholds were determined by using an “up–down staircase” algorithm (Levitt, 1971). For this adaptive psychophysical procedure we used a Matlab toolbox called “Psychoacoustics,” which was downloaded from the website of Padova University Psychology Faculty (Soranzo and Grassi, 2014). A custom m-file was incorporated in this toolbox to create the clicks in the sound card which drove an audiological insert earphone set. A test for a certain base ICI started with an initial bi-directional change of ΔICI = ICI/8, which should certainly be detected and positively responded by the subject. Then the software automatically reduced ΔICI by a factor of 2 and delivered the rate-modulated click train to be judged and responded by the subject. This was repeated until the subject’s response turned to negative. Upon this first reversal of the response, ΔICI was increased in steps with the size of the last step until the response returned to positive. From this second reversal on, the ΔICI was either decreased or increased according to the subject’s response. Upon the 2nd, 3rd, and 4th reversals of the subject’s response the step size was reduced by factors of 1.5, 1.2, and 1.1, respectively, and the test terminated upon the 5th reversal. The geometrical average of the ΔICI values for which the last four reversals occurred was taken as the ICI discrimination threshold.

Sampling rate limitation (80 kHz) of the sound card we employed prevented us from obtaining thresholds for ICIs shorter than 5 ms, because the sample period of 1/80,000 = 0.0125 ms imposed a limit on the minimum ICI change that could be made. This minimum ΔICI is equivalent to a WF of 0.25% in the ICI = 5 ms condition. Since this minimum resolution in WF measurements was already attained by some of the subjects for ICI = 5 ms, this duration was the lower limit of the base ICIs that we could extend our experiments.

Weber fraction for ICIs was calculated by dividing the discrimination threshold measured as described above by the base ICI in that test and it was expressed as the percentage of ICI. It should be noted here that calculation of WF for repetition rate gives the same result as the WF calculated for ICI. Each condition of the experiment (base ICIs of 5, 7, 10, 15, 20, 30, 50, 70, 100, 150, 200, and 300 ms) was tested two times in each subject and the two WF values obtained for each base ICI were averaged. The order of ICIs tested was randomized across subjects. Finally, a graph was obtained displaying the dependence of relative rate discrimination threshold (WF) on ICI as grand average of 10 subjects. Significance of the differences between the WF values in certain ICI ranges was *t*-tested by comparing the average WF levels in the ranges studied. The *p*-values found in the two paired *t*-tests was Bonferroni-corrected to account for multiple comparisons.

## RESULTS

**FIGURE 2:**
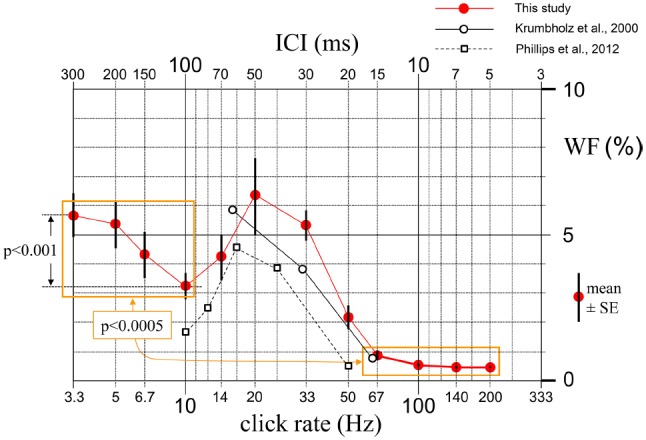
**Mean (10 subjects) and standard errors of the relative rate-change detection thresholds (Weber fractions, WFs) measured for 12 base ICI values between 5 and 300 ms.** Mean of the WFs for the four shortest ICIs proves to be around 10 times smaller than the mean of the WFs for the four longest ICIs (*p* < 0.0005). The decrease of WF at ICI = 100 ms is also significant (*p* < 0.001). Open circles and open squares show the averages of the WF values that can be approximately read from the curves given by Krumbholz et al. (2000) for three subjects and by Phillips et al. (2012) for five subjects, respectively.

The results obtained from the psychophysical tests given to 10 subjects are illustrated in Figure 2 as a plot of the mean WF versus ICI with standard error bars. The plot provided a certain pattern for the relationship between rate discrimination threshold and click rate. Variances around mean WFs for the few highest rates were so small that the error bars are not visible. This must be partially due to the technical limitation of the sound system explained in Section “Materials and Methods.” The WF values for ICI = 5 ms and even those for 7 and 10 ms might not be very accurate because the relatively limited temporal resolution of the sound system was probably not adequate for the subjects with relatively fine discrimination thresholds at these short ICIs. However, this should not pose a problem regarding the aim of the present study, because a lack in temporal resolution would lead to thresholds at low end being overestimated rather than underestimated.

The WFs for relatively longer ICIs was found to be around 10 times larger than those for relatively shorter ICIs. Difference between the mean values of the WFs for the four shortest and four longest ICIs proved to be highly significant [*t*(9) = 7.21, *p* < 0.0005]. Relative discrimination threshold decreased sharply from an average value of around 5% at repetition rates below 20 Hz to about 0.5% at rates above 67 Hz. This drastic transition of the discrimination threshold corresponded to a change in the perceptual quality of the stimuli. Subjects reported that at rates below 20 Hz they perceived periodicity as a fast tapping rhythm, whereas at rates above 50 Hz the perceived quality was a pitch. In other words, it was observed that regular click trains at a rate of less than about 20 Hz sounded like individual regular events, whereas those with rates faster than about 50 Hz merged into a single steady sound, whose pitch depended on the click rate: the faster the rate, the higher the pitch.

Mean WF which was 5.7% initially for ICI = 300 ms dropped to 3.2% at ICI = 100 ms, and this drop was statistically significant [*t*(9) = 5.31, *p* < 0.001]. As ICI was further shortened down to 50 ms, WF increased again, forming a dip at ICI = 100 ms. The individual curves of eight of the 10 subjects had this U-shaped behavior with a minimum around 100 ms (two subjects at 70 ms, five at 100 ms, and one at 150 ms). In order to see if this preference of the ICI around 100 ms among the six ICIs between 50 and 300 ms was statistically significant, a binomial test was conducted. Cumulative probability of having this ratio (eight or more out of 10) by chance was calculated to be *p* = 0.0547, which is very close to significance at α = 0.05.

## DISCUSSION

The two main findings of the present study are (1) a steep change in interval discrimination threshold, thus a discontinuity in WF, for base intervals around 50 ms, and (2) a local minimum in that threshold for click rates around 10 Hz, suggesting a preferred interval of 100 ms in temporal discrimination. These two findings are discussed below together with some relevant data in literature, and the preference at 10 Hz is further discussed in connection with some of the time perception models and its possible link to the electroencephalography (EEG) alpha rhythm.

### WF DISCONTINUITY AROUND ICI = 50 MS

The results, which are in line with those of similar behavioral studies in the literature (Krumbholz et al., 2000; Phillips et al., 2012), show that there is around an order of magnitude difference between the discrimination thresholds for the rates lower than 20 clicks/s and higher than 70 clicks/s. The WF we obtained for an ICI of 200 ms is also very close to the data provided in the work of Grondin (2010) for the same inter-onset interval (IOI) and similar number of repetitions that were used to detect a temporal difference between sequences (5.4% in the present study versus 5.9% in their study). For comparison, the results reported by Krumbholz et al. (2000) and Phillips et al. (2012) are incorporated in Figure 2, where the results obtained over the range of similar stimulation rates in the present study are presented. It is seen in Figure 2 that, leaving aside the rather insignificant discrepancies which may have resulted from methodological differences among studies, the WFs measured by Krumbholz et al. (2000) for three rates and by Phillips et al. (2012) for five rates are fairly close to those measured by us for comparable rates. A dramatic alteration in WF such as the one observed in the 20–70 clicks/s range in both of these earlier studies as well as in the present one, is quite unusual in a single perception mechanism and clearly speaks for the presence of separate mechanisms for processing the click rate at long and short ICIs. This viewpoint is in line with the psychophysical observation made by our subjects that the perceived qualities are different in the two ranges; i.e, fast tapping rhythm versus pitch of a steady smooth sound, respectively.

#### Distinct mechanisms for temporal and spectral discrimination

There are neural imaging studies indicating separate cortical mechanisms for temporal and spectral processing auditory stimuli. For instance the results of the positron emission tomography study of Zatorre and Belin (2001), who examined the response of human auditory cortex to spectral and temporal variation, indicated specialization of the left-hemisphere auditory cortex for rapid temporal processing, and a complementary hemispheric specialization in right-hemisphere belt cortical areas for spectral processing.

**FIGURE 3:**
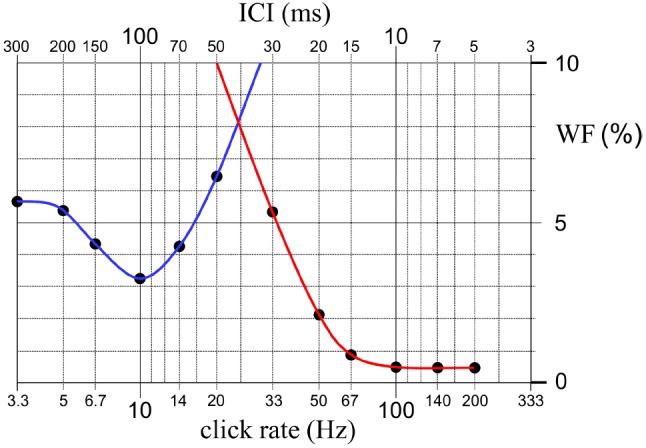
**An illustration showing that the variation of WF as a function of click rate can be modeled as a combination of two curves, one for low and the other for high click rates.** We hypothesize that these two curves, which are drawn by blue and red lines, belong to two separate perception mechanisms for time intervals and pitch, working at long and short inter-onset intervals, respectively. There seems to be a region between the rates of 20 and 33 Hz in which neither of the two mechanisms is as sensitive. This finding probably indicates a transitional gap between the operating ranges of the presumed temporal perception and pitch perception mechanisms.

It is illustrated in Figure 3 that the above-explained behavior of the WF versus click rate function can be modeled as a combination of two curves, one for low and the other for high click rates. We hypothesize that these two curves belong to two separate perception mechanisms for time intervals and pitch, respectively. There seems to be a region around 20 Hz in which the rate discrimination threshold is elevated to a maximum. Such a maximum has also been reported in the recent work of Phillips et al. (2012) who studied human sensitivity to rate alteration in 25-click trains with ICIs between 20 and 100 ms using wideband clicks. They describe maximal WFs for ICIs around 40–60 ms, which exactly coincides with the ICI for which the WF displays a maximum in the present study (i.e., 50 ms).

It is to be noted that this region, in which both of the presumed time and pitch mechanisms are relatively insensitive to rate alterations, coincides with the range of repetition rates in which an initially high-frequency click train perceived as a continuous steady sound starts to be described by the subjects as a fluttered sound as the click rate is decreased. Based on these psychophysical observations, we speculate that, in the low repetition rate region a temporal perception mechanism, which analyses time intervals between peaks in the neural activity pattern, is active; whereas in the higher repetition rate region a pitch perception mechanism, which analyses the spectral features of the sound coded tonotopically in the peripheral and central mechanisms of the auditory system, takes over. However, as schematized in Figure 3, the overlap between the operating ranges of the two postulated mechanisms seems to leave a transitional gap around 20 Hz where neither of the two mechanisms is as sensitive. This interpretation was also made by Phillips et al. (2012) based on their observation of maximal WFs for ICIs around 40–60 ms.

We should also mention here the work of Warren and Bashford (1981). They repeated Gaussian noise segments at different rates and asked the subjects to choose the rate corresponding to their lower limit for pitch, and also to select the rate within the pitch range corresponding to the transition from a smooth, homogenous sound to a rough, pulsed sound. They reported two transition zones, one based on a judgment of pitch/infrapitch boundary at around 20 Hz and the other one based on a judgment of smooth/pulsating boundary at around 70 Hz. As illustrated in Figure 3, these two boundaries almost exactly coincide, respectively, with the lower limit of the WF curve that we presumed to be associated with pitch mechanism, and the click rate at which the differentiation threshold of the same mechanism reaches its ultimate minimum. Based on these psychophysical observations, 20–70 Hz range of the curve can be associated with a transition from perception of the temporal or periodicity pitch to perception of the spectral or place pitch.

#### Possible neural basis for the WF discontinuity around ICI = 50 ms

The perceptual discontinuities observed in rate discrimination threshold should of course have some neural basis. Indeed, in the auditory nuclei at various subcortical levels there exist a variety of neurons and neuronal circuits coding the periodicity and rate of a repetitive sound (Langner, 1992). However, as it is the final stage of neural processing before an event is perceived, we rather focus here on the cortex to find some neural correlates for such discontinuities. It was reported by Lu and Wang (2000) that majority of the neurons they studied in the cat auditory cortex (area AI) exhibited significant stimulus-synchronized discharges at long ICIs. As the ICI was shortened, however, the synchronization began to degrade and they were able to record no significant response synchrony at ICIs shorter than 40–50 ms for most of the units they studied. At such very short ICIs, on the other hand, they found a population of neurons that were able to signal ICI changes by their discharge rates, suggesting a complementary implicit rate code that replaces the explicit temporal code. These physiological findings are suggestive of the neural correlate of the observed perceptual change from a series of discrete auditory events to a single sustained sound as the ICI decreases from 50 to 20 ms.

### PREFERENCE OF 100 ms IN TEMPORAL DISCRIMINATION AND ITS POSSIBLE CONNECTION TO THE EEG ALPHA RHYTHM

Another notable finding of the present study is the significant decrease of the relative threshold (WF) as the click rate is reduced from 20 to 10 clicks/s. This finding is in line with the work of Michon (1964) who measured listeners’ ability to detect tempo differences for ICIs and reported a minimum WF of ca. 1.0% for an ICI around 100 ms. The difference between the WF values reported in his and our studies probably results from the fact that relatively long sequences and very well practiced subjects were used in his work. A similar decrease in WF at 100 ms ICI was also reported by Phillips et al. (2012), although their data were not extended enough to show if this drop corresponded to a minimum (see the approximately averaged data shown by open squares in Figure 2).

In fact, there are a number of studies reporting not a decrease but an increase in temporal discrimination threshold as the IOI is reduced to 100 ms (e.g., Hirsh et al., 1990; Drake and Botte, 1993; McAuley and Kidd, 1998). This discrepancy is probably due to methodological differences, considering the fact that the precision of interval discrimination tests depends on many different factors, in particular the method used and the physical characteristics of the events marking the intervals, as also stated by Drake and Botte (1993). In some of the time interval discrimination studies a variation of a two-duration paradigm is used. Subjects are presented two filled or empty intervals and are asked to choose the longer (or shorter) of the two. In some others, one of the intervals in an otherwise regular sequence of beats is made shorter or longer and subjects are asked if they notice the irregularity. In still some others, two regular sequences of beats separated by a certain gap are presented for comparison. To construct the sequences to be compared, time intervals are bounded by a pair of clicks in some studies and by tone bursts with various durations in some others. Methodological differences among this kind of studies may lead to quite discrepant results. It is therefore difficult to discuss why some of the results of the present study may be different than their results. Nonetheless, we have selected a few representative studies and discussed below why our finding that the relative threshold at around ICI = 100 ms is lower than the threshold for longer ICIs could be in disagreement with some previous reports.

For instance, in the work of Drake and Botte (1993), in which isochronous sequences consisted of the same number of stimuli for all the IOIs studied, the “multiple looks” factor described by the authors must not have differentially affected the listeners’ tempo discrimination thresholds for different rates. In our study, however, because we fixed the length of each isochronous sequence at 1.5 s, a sequence included 15 intervals of 100 ms, whereas only five intervals of 300 ms could fit into a sequence. This may have provided a “multiple-interval” advantage (McAuley, 2010) for the former, leading to a lower tempo differentiation threshold for the IOIs around 100 ms than for longer IOIs. Moreover, in the works of Drake and Botte (1993) and McAuley and Kidd (1998), sequences were composed of 440-Hz, 50-ms tones. For IOI = 100 ms, the sequences to be compared were both square-wave amplitude-modulated (AM) tones with identical pitch. This similarity between the two sequences compared may partially mask their temporal dissimilarity, making it difficult to notice the difference between them and leading to higher relative just noticeable differences (JNDs) for that IOI. At relatively longer IOIs, on the other hand, the sequences of discrete tone bursts are perceived as a tempo rather than an AM tone, and differences between their tempi can be detected more neatly without being confounded by spectral similarity of the tones. The same argument applies to the work of Hirsh et al. (1990) in which standard isochronic series of a number of 1000 Hz, 20 ms-tones separated by IOIs of 200, 100, or 50 ms were used and the discrimination threshold for IOI = 100 was found to be lower than the threshold for IOI = 50 ms, but higher than the threshold for IOI = 200 ms. Because 0.1 ms-clicks rather than tone bursts were used in the present study to construct the sequences, there was no such tonal similarity effect which would have increased the WF values for relatively shorter IOIs. Interestingly, also in the work of Michon (1964) where a minimum rather than an increase was reported for IOI = 100 ms, the intervals to be compared were bounded not by tone bursts but click trains.

The observed preference of ICI = 100 ms for which the tempo discrimination threshold displays a minimum in the present study corresponds, interestingly, to the period of the most prominent oscillatory activity in EEG, namely, the 8–12 Hz alpha rhythm. This coincidence may imply a connection of the alpha rhythm with detection of a change in IOIs in a sequence, which can be based on the following three main approaches used in models of interval timing.

#### Possible alpha connection considering clock-counter approach

A speculation on a possible function of the alpha rhythm in interval timing would be based on a clock-counter device as presumed in a very influential theory in timing research (Wearden and Grindrod, 2003). The alpha rhythm would act as the pacemaker of the internal clock. It is not difficult to imagine that a clock mechanism based on accumulation of pacemaker pulses can only work for intervals that are equal or longer than the intervals of the pacemaker, and its performance will sharply deteriorate for intervals which are shorter than even a single pacemaker period. The rather steep elevation of the threshold for detecting ICI alterations at click rates higher than 10 Hz may be because of such a sharp decline in the performance of the presumed internal clock. Actually, it is this sharp increase and the following sharp decrease for faster rates that make us think the “WF–click rate” relationship as being composed of two functions each belonging to a different mechanism, as illustrated in Figure 3.

#### Possible alpha connection considering entrainment approach

Another explanation for the minimum in discrimination threshold at ICI = 100 ms would be based on an alternative model of tempo discrimination involving an entrainment approach (McAuley and Jones, 2003; McAuley, 2010). In this model, a sequence generates an internal rhythm, and the onset times expected according to this internal rhythm are compared with the onset times of the actual beats. The temporal discrepancy between the two is perceived as an interval mismatch. Concerning rhythm generation, the brain is known to have susceptibility around 10 Hz, which reveals itself as abundant EEG oscillations in the alpha frequency range. This susceptibility may be the mechanism underlying the 100 ms preference we observed in our experiments for the following two reasons:

(a) Because entrainment models assume a gradual generation or correction of an internal rhythm, the sequences used in discrimination threshold tests should be long enough as to allow for an internal rhythm to develop. This condition is more satisfied in the present study than those reporting not a decrease but an increase in threshold for IOI = 100 ms. For instance, in the work of Hirsh et al. (1990), the maximum length of the sequences presented to their subjects for comparison was 600 ms for 100 ms IOIs. In the present study, on the other hand, each of the six consecutive isochronous sequences evaluated by the subjects was 1.5 s long, and the subjects enjoyed the advantage of hearing five consecutive tempo alterations in a single trial. Furthermore, there was no gap between the sequences with slightly different tempi, allowing for an efficient comparison between the onset times that are expected according to the internal rhythm generated by the former sequence and the onset times of the beats in the latter sequence.
(b) In many differential threshold measurements in literature, only the one or two of the stimuli in the sequence is shifted in time. For instance, in the work of Hirsh et al. (1990), subjects’ task was to detect an asynchrony or deviation in the time position of only one stimulus in an otherwise isochronous sequence. In such cases the average stimulus rate of the standard and comparison sequences, and thus the average timing of the beats of the internal and actual rhythms, would become very close to each other. This is not a favorable condition for an entrainment mechanism working on the basis of timing mismatch. Therefore, susceptibility of the brain regarding alpha rhythm generation will not be instrumental, and thus 100 ms-preference in tempo discrimination ability may not be observed. In the present study, on the other hand, six consecutive 1.5-s isochronous sequences of clicks with two alternating rates were presented to the subjects and their task was to detect the abrupt changes in rate at the borders of the successive sequences.

Considering the two arguments discussed above, the conditions for an entrainment-based mechanism to function appear to be more satisfied in the present study than those reporting not a decrease but an increase in threshold for IOI = 100 ms. From this point of view, the decrease in tempo discrimination threshold observed at intervals around 100 ms in the present study, which is in contrast with other data reported in literature for relatively short or asynchronous sequences, may be taken as a finding supporting the entrainment model of tempo discrimination.

#### Possible alpha connection considering coincidence detection approach

One may also relate the finding of relatively lower ICI change threshold in the alpha frequency range to another class of interval timing models which propose that the detection of coincident neural activity encodes the duration of events. The model proposed by Matell and Meck (2000), for instance, is based on decision neurons which become active only when a particular set of the oscillating neurons are coactive. They suggested that cortical–striatal–thalamic loops may provide the neuronal circuitry needed in a mechanism of interval timing based on coincidence detection. Although there might not be a direct connection of the alpha rhythm to the oscillations mentioned in their model, the period of this rhythm (ca. 100 ms), which is believed to be generated by the thalamo-cortical circuits of the brain (Lopes da Silva et al., 1974; Bhattacharya et al., 2013), may suggest the presence of adequately long delay lines or loops that are needed by a coincidence-detection mechanism to function as an interval timer. If a connection between the alpha rhythm and thalamo-cortical loops with long delays is assumed, and the fact that 10 Hz-oscillations are the most prominent rhythm in EEG is considered, one may expect that relatively larger number of delay lines are devoted to the time range around 100 ms. A finer temporal resolution thus provided in this range of time intervals would be an alternative explanation for the relatively low ICI discrimination threshold observed at around 100 ms.

### FOR FUTURE RESEARCH

Considering the recent literature on the importance of the auditory cortex for temporal discrimination not only of auditory but also of somatosensory and visual stimuli (Bolognini et al., 2009; Kanai et al., 2011), it should be quite interesting to know if a similar preference exists also for intermittent visual and vibratory tactile stimuli at repetition rates around 10 Hz. Results of such a study may contribute to the discussion on the competing ideas of a “single amodal” and “multiple modality-specific” temporal mechanisms (Wiener et al., 2011).

It has been pointed out above that the decrease in tempo discrimination threshold observed at intervals around 100 ms in the present study but not in most of the studies in literature, may be taken as a finding supporting the entrainment model of tempo discrimination (McAuley and Jones, 2003). This view point is based on a hypothesis that long and isochronous sequences should be more effective than short and/or asynchronous sequences in generating or correcting of an internal rhythm through entrainment. It would be interesting, therefore, to test this hypothesis by comparing, in a single study, the rate discrimination thresholds for the two types of sequences at IOIs around 100 ms.

It should also be interesting to study the correlation between the individual alpha frequency and the dip frequency of that individual around 10 Hz for testing the viability of our speculation that the alpha rhythm would have a connection with time perception. Another interesting question would be to ask if the amount of individual alpha power correlates with the tempo differentiation performance of that subject for click frequencies around 10 Hz. These two follow-up questions would be the subject of future studies in which the subjects’ EEG will be recorded and analyzed together with their psychophysical data.

## AUTHOR CONTRIBUTIONS

Pekcan Ungan conceived the study, designed the experiments and experimental setup, performed most of the experiments, collected the data, and wrote the manuscript. Suha Yagcioglu designed the experiments and experimental setup, wrote the codes to implement the psychophysical tests, analyzed the data, and revised the manuscript critically.

### Conflict of Interest Statement

The authors declare that the research was conducted in the absence of any commercial or financial relationships that could be construed as a potential conflict of interest.
